# Motivations for Abortion or Continuation of an Unwanted Pregnancy: A Scoping Review of the Global Literature

**DOI:** 10.1111/psrh.12293

**Published:** 2025-01-22

**Authors:** Wieke Y. Beumer, Annemarie Y. A. M. Reilingh, Eline Dalmijn, Tessa J. Roseboom, Jenneke van Ditzhuijzen

**Affiliations:** ^1^ Epidemiology and Data Science Amsterdam UMC Location University of Amsterdam Amsterdam The Netherlands; ^2^ Obstetrics and Gyneacology Amsterdam UMC Location University of Amsterdam Amsterdam The Netherlands; ^3^ Amsterdam Reproduction and Development Amsterdam the Netherlands; ^4^ Amsterdam Public Health Amsterdam The Netherlands; ^5^ Public and Occupational Health Amsterdam UMC Location Vrije Universiteit Amsterdam Amsterdam The Netherlands; ^6^ Research Department Fiom 's‐Hertogenbosch The Netherlands; ^7^ Interdisciplinary Social Science, Social Policy and Public Health Utrecht University Utrecht The Netherlands

**Keywords:** abortion, pregnancy decision making, scoping review, unwanted pregnancy, women's health

## Abstract

**Context:**

The aim of this scoping review was to provide an overview of recent studies in peer reviewed journals investigating self‐reported motivations to have an abortion or to continue an unwanted pregnancy in different countries and settings, including both qualitative and quantitative results.

**Methods:**

We searched for English language publications published between 2008 and 2023 indexed in four scientific databases. We included studies if they captured people's own motivations for abortion and/ or for continuing an unwanted pregnancy.

**Results:**

Of the included 19 studies, all focused on abortion, and four also included motivations to carry an initially unwanted pregnancy to term. Motivations for abortion often related to family planning (e.g., complete family, no desire for children, not the right time), the relationship with the person involved in the pregnancy, and life or material circumstances (such as financial resources, housing or future plans), and sometimes with stigma, shame, or expected negative reactions. Motivations to continue an unwanted pregnancy were having a supportive partner and personal beliefs about the pregnancy. Despite different settings, different methods, and methodological limitations, studies showed similar multifactorial and interrelated motivations in decision making around unwanted pregnancies.

**Conclusions:**

This research showed that in different places throughout the world multiple interrelated motivations play a role in a decision to have an abortion or to continue an unwanted pregnancy. The findings mainly provide insight into retrospective explanatory accounts, which may be biased because respondents may feel the need to justify their choice. Future research should discontinue asking people to rationalize unwanted pregnancy decisions.

## 1 Introduction

Unintended pregnancy is a common phenomenon worldwide, and it is estimated that around 50% of unintended pregnancies are carried to term [1]. People can experience an unintended pregnancy as unwanted, which may result in having an abortion [2]. In many countries, people can legally choose abortion, but in other countries, abortion is prohibited or restricted [3]. Abortion rates do not exhibit a direct correlation with abortion laws [4]. However, in countries where abortion is restricted it is typically only legally available under specific circumstances. Despite these restrictions, abortion is often accessed clandestinely, both safely and unsafely. The “reasons” for abortion are often the topic of political and public debates. In these debates, the same questions are frequently raised: why do people have abortions? And why do others choose to continue an unwanted pregnancy? The current study focuses on motivations to have an abortion or to continue a pregnancy that is not only unplanned or unintended, but also experienced as unwanted at some point. Although previous studies and policy often focused on “reasons”, we use the term “motivations” throughout this manuscript to better capture both rational and emotional aspects of decision‐making.

People's motivations in decisions about unwanted pregnancies are particularly personal and depend on the context in which the decision is made [5]. While large‐scale cohort studies can theoretically describe the correlates of unintended pregnancy and the subsequent decision to have an abortion or to continue an unwanted pregnancy, they do not reveal the specific motives that actually drive this decision‐making process. To gain more insight into the decision‐making process, womxn's^1^ own accounts of the factors most important to them are needed [6].

Two prior review studies, published in 1998 and 2009, captured self‐reported motivations people give for abortions [6, 7]. Both concluded that motivations for abortion are often complex and contingent. According to the authors of these review studies, people often consider their own needs and desire to have children, the role of the partner involved in the pregnancy and have a sense of responsibility toward existing children and the potential child. They also consider the material context in which the pregnancy takes place, such as inadequate financial resources, employment status and lack of space in the house.

The most recent review study published in a peer reviewed journal dates back 15 years, and included both quantitative and qualitative results [6]. The aim of the current study is to provide an extended and updated literature review, while adding a critical appraisal of methodological quality of included studies. Kirkman's review [6] focused on high‐income countries only, yet a worldwide approach may add valuable insight about the potential universality and contextual differences in motivations that are mentioned. Both previous review studies focused on motivations for abortions only. Hence, there is a lack of insight into experiences of people who decide to continue an unwanted pregnancy. This is important since approximately half of unwanted pregnancies worldwide are carried to term [1]. The current review study aims to fill these research gaps.

## 2 Methods

### 2.1 Protocol and Preregistration

The Dutch Ministry of Healthcare, Wellbeing and Sports requested the investigation of circumstances that contribute to abortion or continuation of unwanted pregnancies, which led to the formation of the research question. The Ministry had no role in the development of the research design, methods, the execution, and reporting of the research. This scoping review was part of a larger research project in which we collaborated with diverse Dutch expertise centers (such as Fiom, the Dutch expertise Centre on unwanted pregnancies).

Authors W.Y.B., A.Y.A.M.R., J.v.D., and E.D. developed the protocol and search strategy, based on the methodological guidelines of Arksey and O'Malley, providing a framework for conducting scoping reviews to map key concepts, evidence types, and research gaps [8]. We preregistered the protocol at OSF, an open science platform (osf.io/gn8cp).

### 2.2 Search Strategy

We conducted a scoping review of existing literature in peer reviewed journals as indexed in four relevant computerized databases (Scopus, PsycInfo, Web of Science and PubMed). We searched in these four databases to cover publications in both medical and social sciences. The search strategy per database are included in Tables S1a to S1d. We included studies with a publication date limited to 2008–2023 to provide an update on the review of Kirkman et al., which included studies published before 2008 [6]. We did not add terms related to adoption as a pregnancy outcome, since this option is hardly ever considered by people with an unwanted pregnancy within the gestational limit for abortion [9, 10]. Furthermore, this would entail adding a whole new and highly specialized field of research, outside of the scope of the current research project.

We used several inclusion criteria in the current review study (Table 1). We purposefully included all study designs that came up (e.g., interviews, cohort studies, questionnaires, etc.) except review studies. Unlike Kirkman et al. [6], we did not exclude studies in lower and middle income countries to have a larger selection of studies for this review, but also because we wanted to investigate to what extent context is relevant for the outcomes.

**TABLE 1:** Inclusion and exclusion criteria.

Inclusion criteria	Exclusion criteria
Focused on unintended pregnancies (unintended, unplanned and/or unwanted) Carried to term (parenting) Terminated (abortion) Or both	Not focused on unintended pregnancies
Included topics about unintended pregnancy decision making, circumstances, explanations, reasons and/or motives	Studies focused on predicting people's risk of experiencing an abortion based on sociodemographic characteristics
Focused on perspectives of people who experienced an unintended/unplanned/unwanted pregnancy themselves or as a “partner” (i.e., someone involved in the pregnancy)	Focused on perspectives of people without specific unintended pregnancy experience (i.e., clinicians, studies focusing on the hypothetical attitudes toward abortion from people without actual experiences with unintended pregnancy)
Primary studies	Opinion, dissertations, student theses, conference presentations, review studies or discussion papers
Written in English	Language other than English
Published between 2008 and 2023	Published before 2008

**FIGURE 1:**
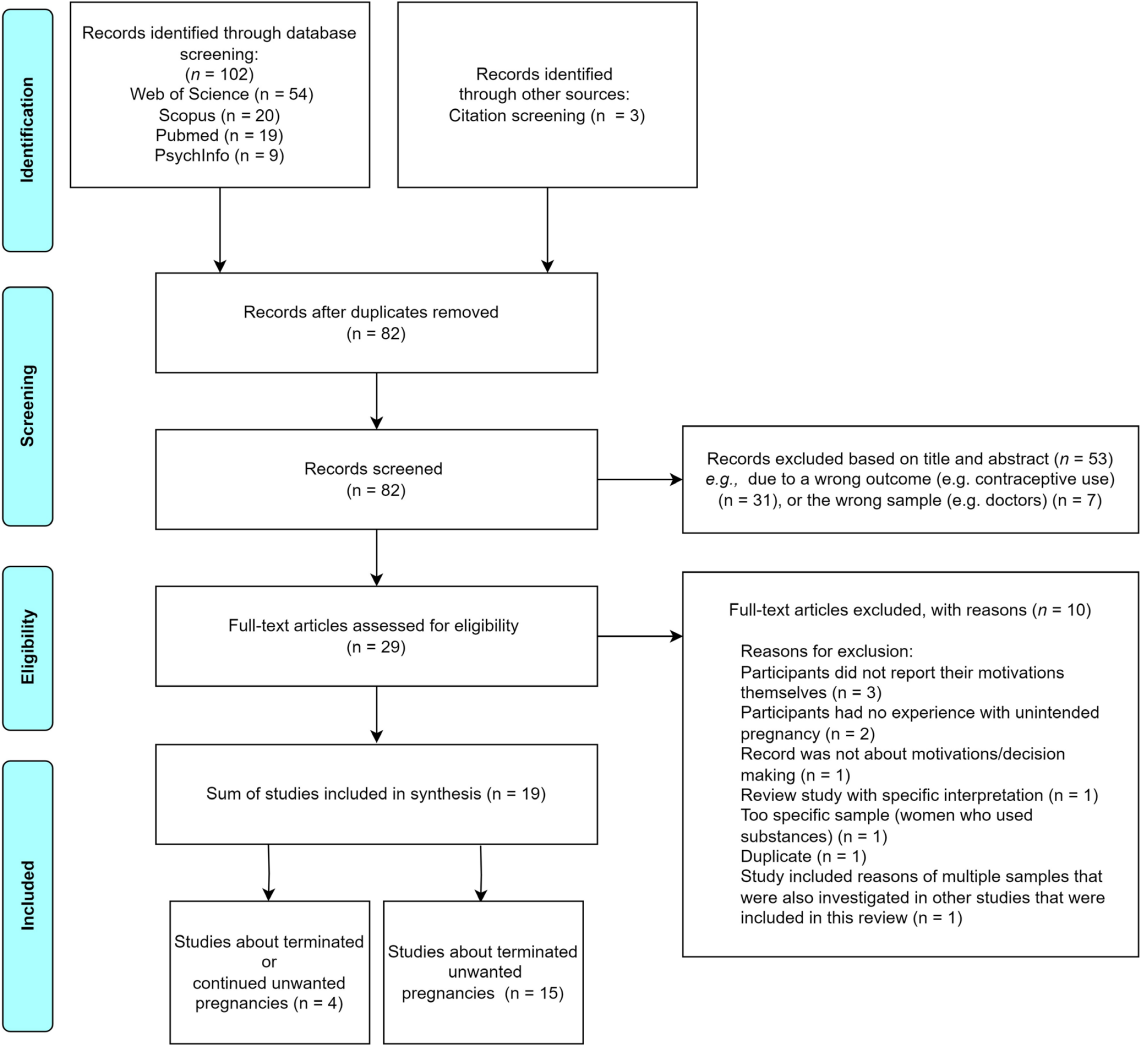
PRISMA flowchart of the search and selection process.

We carried out the selection procedure in steps, following the Preferred Reporting Items for Scoping Reviews (PRISMA‐ScR) [11] (see Figure 1). After completing the search in the four databases and removing duplicates (using EndNote [12]), we selected papers based on their title and abstract using Rayyan [13], an online application for conducting literature reviews. W.Y.B. screened all articles. A.Y.A.M.R., E.D., and J.v.D. each performed a second screening on a sub‐selection of the articles, resulting in dual independent screening of all retrieved articles. Additionally, we screened reference lists of included articles, and read full text versions of the selected articles. Again, W.Y.B. screened all selected articles and A.Y.A.M.R., E.D., and J.v.D. screened a sub‐selection so that all selected articles were double‐screened. We documented screening results manually in MS Excel, and compared and discussed the double‐screened results among during weekly meetings. Lastly, we critically appraised the included studies, using the checklists of the Critical Appraisal Skills Programme (CASP) for qualitative and cohort studies [14, 15] (Tables S2a and S2b). The CASP checklists contain structured questions that assess the methodological quality, relevance, and rigor of studies. Key components include evaluating the clarity of research aims, appropriateness of methodology, recruitment and data collection methods, consideration of ethical issues, thoroughness of data analysis, and the applicability and value of findings to practice or further research.

### 2.3 Analysis

We analyzed the included articles following an iterative process. First, we identified and tabulated the research aims, outcomes, samples, and country of origin per included study, as well as methodological characteristics. Second, we started extracting and categorizing the motivations for abortion or for continuing an unwanted pregnancy based on the categories used in the review by Kirkman et al. [6]. During this process, we relabeled and reorganized the initial categories to reduce overlap which could not be avoided completely due to the interrelated nature of motivations. Decisions were made based on consensus within the team and aimed to optimally capture underlying themes of lived experiences. For instance, Kirkman et al. [6] used the category “woman‐focused reasons”, which we decided to divide into motivations related to family planning/ desire to have a child, life circumstances and personal beliefs.

The research team consisted of white female researchers born in the Netherlands, with quite similar cultural backgrounds. To sensitize ourselves and create awareness on our position, we frequently reflected on our positionality during the discussion of our findings. We further discussed our findings with an advisory committee of the research project that this study is part of. This committee consisted of experts in the field of (reproductive) healthcare, social policy research, health ethics, and a member of the interest group for contraception and abortion.

## 3 Results

The search generated 82 unique articles after duplicate removal (Figure 1). We excluded articles that failed to meet the inclusion criteria (Table 1). The most common reason for exclusion was that articles focused on motivations for using a certain contraceptive method (post‐abortion), which was not the focus of the current literature review. We excluded one study to avoid duplication, as it contained data from another study in the selection.

We finalized the selection, which included 19 papers for quality assessment. We began by categorizing articles into interview‐based and survey‐based studies to facilitate comparison in the CASP appraisal [14, 15], but we found that the boundaries between these categories were not strictly quantitative versus qualitative: some survey studies used open‐ended questions which were coded into themes, and some interview studies were highly structured. There were also two studies based on national population cohort survey data [16, 17]. Therefore we decided not to categorize articles in quantitative versus qualitative design.

Characteristics of the selected studies, such as study aims, methods, and setting (i.e., country and legal status of abortion at the time the study was conducted [18]), are displayed in Table 2. According to the classification of the World Bank [19], 11 were set in high income countries (HICs), five in upper middle income countries (UMICs), and three in low middle income countries (LMICs) (Table 2).

**TABLE 2:** Characteristics of published studies assessing motivations for abortion or continuation of an unwanted pregnancy (*n* = 19).

Reference	Context	Sample and study	Study aim	Methods	Operationalization of motivations
Bell et al. [30]	UK: HIC Abortion allowed on request (gestational age limit of 24 weeks)	28 young womxn (13–18 years); 19 who had an abortion, nine who continued an unwanted pregnancy. Also a comparison group of womxn who were never pregnant (23 womxn)	Investigating teenagers' future orientations in relation to deciding what to do with their unwanted pregnancy	In‐person orally administered survey, partly closed/open questions	Participants were asked what they thought about when making the decision to have an abortion or to continue the pregnancy, responses were coded by the researchers
Biggs et al. [35]	USA: HIC Federal system: law varies at state level, with restrictions in some states; study was conducted before Roe v Wade was overturned in 2022	725 womxn (15–46 years) who had an abortion, 231 who were denied an abortion from the Turnaway Study	Describing reasons for wanting an abortion	Structured telephone interviews, partly closed/open questions	“What are the reasons you decided to have an abortion?” followed by a prompt asking for any other reasons until respondent says that is all. The second question: “What would you say was the main reason you decided to have an abortion?”
Biney and Atiglo [16]	Ghana: LMIC Abortion allowed to save a person's life	552 womxn (15–49 years) who participated in the Ghana Maternal Health Survey in 2007 and had an abortion in the past 5 years	Understanding reasons for abortion	In‐person orally administered survey, partly closed/open questions	“What was the main reason you had an abortion?” The answers were coded into: (1) financial, (2) study/career, (3) birth spacing, (4) lack of social support, (5) health, and (6) other reasons. Authors state “although the respondents may have had several reasons for terminating (…), they were asked to provide the main reason”
Brauer et al. [28]	The Netherlands: HIC Abortion allowed on request (gestational age limit of 24 weeks)	109 womxn (18–45 years) with unwanted pregnancies; 40 continued the pregnancy, 69 had an abortion	Understanding the decision‐making process of womxn with an unintended or unwanted pregnancy	In‐person in‐depth interviews using a topic guide	From the topic list: Motivation for the decision (reasons and feelings for/against carrying the pregnancy to term and abortion, decision difficulty)
Chunuan et al. [27]	Thailand: UMIC Abortion allowed to save a person's life; study was conducted prior to significant expansion of abortion laws in 2021	402 womxn (mean age 27) who were hospitalized after an abortion: 2 had a legal abortion, 238 had a spontaneous abortion, and 143 had an illegal abortion	Understanding reasons for illegal abortion	Survey questionnaire filled out by participant, closed questions	By means of a checklist (topics related to (1) family problems, (2) financial, (3) social), specific questions not described
Ekstrand et al. [25]	Sweden: HIC Abortion allowed on request (gestational age limit of 18 weeks)	25 womxn (16–20 years) who had a recent abortion	Understanding the circumstances surrounding (decision‐making in) teenage pregnancy	In‐person in‐depth interviews using a topic guide with open‐ended questions	Exact question not described. Open‐ended question about “experiences with decision‐making process”
Hosseini‐Chavosh et al. [21]	Iran: UMIC Abortion allowed to save a person's life; study was conducted before further restrictions on abortion laws in 2021	40 womxn (15–54 years) who had an abortion of an unwanted pregnancy who were selected from the Iran Low Fertility Survey	Understanding the decision‐making process and the reasons for abortion	In‐depth interviews	Not described
Jones et al. [24]	USA: HIC Federal system: law varies at state level, with restrictions in some states; study was conducted before Roe v Wade was overturned in 2022	38 womxn (16–41 years) who had an abortion	Investigating in what way feelings of responsibility and care for existing and future children influence the decision to have an abortion	In‐person in‐depth semi‐structured interviews at abortion clinics	Exact question not described. It is mentioned that the interviews revolved around the reasons for choosing to have an abortion. Authors state: “although not in the interview guide, the topic of motherhood systematically emerged”
Kirkman et al. [36]	Australia: HIC Abortion allowed on request (gestational age limit vary per state)	60 womxn (16–38 years) with unwanted pregnancies; five continued the pregnancy, 55 had an abortion. Three categories of womxn were interviewed: (1) aged 16–18; (2) living in rural and regional areas; and (3) with a second trimester abortion.	Understanding womxn's perspectives on considering abortion (motivations for continuing the pregnancy are not the focus of this study)	In‐depth telephone interviews	The interview included four questions designed to prompt discussion about experiences and reflections on motivations for unwanted pregnancy decisions. To avoid conveying a message that womxn had to defend themselves, womxn were at no time asked why they had considered or undergone abortion, but womxn were invited to talk about their experiences in a free‐flowing manner
Mahanaimy and Moseson [29]	USA: HIC Federal system: law varies at state level, with restrictions in some states; study was conducted before Roe v Wade was overturned in 2022	25 womxn (18–30 years) with at least one unintended pregnancy at or before age 25, irrespective of the outcome of the pregnancy	Understanding young people's decision‐making during unintended pregnancy, with a focus on how social support	In‐depth semi‐structured interviews, in‐person or telephone	“Can you describe the process of how you decided to terminate/carry the pregnancy to term?” Subsequently, specific questions were also asked about social support
Makenzius et al. [32]	Sweden: HIC Abortion allowed on request (gestational age limit of 18 weeks)	798 womxn who had an abortion (14–49 years), recruited at abortion clinics	Identifying factors associated with experiencing multiple abortions	Survey questionnaire filled out by participant, partly closed/open questions	Specific question not described, open‐ended question about “reason for requesting abortion”
Motaghi et al. [22]	Iran: UMIC Abortion allowed to save a person's life; study was conducted before further restrictions on abortion laws in 2021	33 womxn who had an illegal abortion and 19 womxn with an unwanted pregnancy (age reported in categories)	Understanding the causes of illegal abortion	Semi‐structured interviews, answers were coded and quantified in categories	Not described
Pereira et al. [33]	Portugal: HIC Abortion allowed on request (gestational age limit of 10 weeks)	422 womxn (of which 248 adolescents) who had an abortion (mean age around 22 years)	Investigating abortion decision‐making processes	Survey questionnaire filled out by participant, partly closed/open questions	List of 25 reasons for abortion which are rated on a 4‐point scale from 1 (not at all important) to 4 (very important), based on the Reasons for Abortion List (RAL; Broen et al.), including an open answer option
Pestvenidze and Stray‐Pedersen [17]	Georgia: UMIC Abortion allowed on request (gestational age limit of 12 weeks)	Registration data of 2054 womxn who had an abortion (25–34 years) from the Georgian Reproductive Health Survey 2010	Investigating differences among womxn in background characteristics and their reasons for abortion	Survey questionnaire filled out by participant, partly closed/open questions	Participants could choose one reason from a list of reasons: pregnancy was life or health threatening, risk of birth defects, socioeconomic reasons, did not want more children, spacing next pregnancy, partner did not want children, did not have a partner/husband, other
Ranji [31]	Iran: UMIC Abortion allowed to save a person's life; study was conducted before further restrictions on abortion laws in 2021	2705 womxn, of which 17% had an illegal abortion (mean age around 31 years)	Investigating the prevalence of illegal abortion in Iran and understanding which factors influence the decision of abortion	Orally administered survey, partly closed/open questions	Open‐ended question about reasons for abortion (specific question is not described), these reasons were categorized by the authors into 10 items
Rehnströhm Loi et al. [23]	Kenya: LMIC Abortion allowed to preserve health	15 interviews with a total of nine womxn (19–32 years) who recently had an abortion and received post‐abortion care	Investigating abortion decision‐making processes	In‐person in‐depth semi‐structured interviews using open‐ended questions and suggestions for probing	Not described, it seems that motivations emerged as a category
Rowe et al. [34]	Australia: HIC Abortion allowed on request (gestational age limit vary per state)	Registration data of the comprehensive electronic database of the Pregnancy Advisory Service (PAS) of 3598 womxn (13–49 years)	Investigating the demographic and psychosocial circumstances of women considering abortion	An audit was completed for each consultation with womxn contacting the PAS	The main reason for considering abortion was selected from a drop‐down list (only one option possible)
Serret and Pairo [20]	Spain: HIC Abortion allowed on request (gestational age limit of 14 weeks)	25 nulliparous womn (25–34 years)	Understand why womxn want abortions	In‐person in‐depth semi‐structured interviews	Topic guide structured around each stage of pregnancy decision‐making process, probing for motivations not described
Thapa et al. [26]	Nepal: LMIC Abortion allowed on request since 2002 (gestational age limit of 12 weeks)	672 womxn (16–46 years) who had a recent abortion	Understanding the profiles of womxn seeking an abortion, including the situation resulting in unwanted pregnancy and reasons for the abortion	Orally administered survey, partly closed/open questions	Multiple response item, exact question not described

*Note:* Reported legal situation is at the time the study was conducted [18]; countries income is based on the World Bank classification [19].

### 3.1 Critical Appraisal Results

Most quality concerns related to the way the “motivations” (mostly described and studied as “reasons”) were measured in included studies. Five out of ten included interview studies lacked information about the ways motivations were discussed in the interviews [20, 21, 22, 23, 24]. Another quality issue in five included interview studies was that researchers failed to consider the relationship between themselves and participants (i.e., the research did not report anything about whether they critically examined their own role, potential bias and influence) [20, 21, 22, 24, 25] (Table S2a). In two out of nine questionnaire studies, researchers did not provide information about the questions or questionnaires they used to measure motivations [26, 27] (Table S2b). Despite these quality concerns, we found all 19 articles adequate according to PRISMA standards for scoping reviews (based on clear research aims, rigor in study designs and clear reporting of results) [11], and included these in the current scoping review.

### 3.2 Variation Between Included Studies

All selected studies focused on abortion. Only four out of the 19 selected studies also investigated pregnancies that were initially unwanted, but carried to term [20, 28, 29, 30]. No study solely focused on the decision to continue an unwanted pregnancy.

The included studies were conducted all over the world in very different settings (Table 2). The considerable variation in countries reflected not only in structural factors such as legality and access to abortion services, but also in potentially different normative views on abortion and sex outside of marriage. Researchers from HICs were involved in the study design or analyses of some of the included studies in LMICs and UMICs [21, 23, 26]. This may have influenced findings related to normative or cultural differences in the respective studies.

Some selected studies examined a specific group of individuals (Table 2), such as young people with unwanted pregnancy (< 25 years) [25, 29, 30]. Other studies specifically focused on people who had an illegal abortion [22, 27, 31]. One study focused on mothers [24], and another one on individuals with repeat abortions [32]. The included studies focused primarily on womxn with an unwanted pregnancy. No study reported gender identity of participants or the inclusion of people who do not identify as female.

Nine included studies utilized primarily quantitative methods (Table 2), that is, surveys based on interviews or questionnaires [16, 26, 27, 30, 31, 32, 33], but also patient registry data [17, 34]. Some of these used a checklist of potential motivations, where participants indicated which motivations applied to their decision, including an “other” option for motivations not listed [17, 26, 33]. In two studies, data were based on registry data surveys in which only one main motivation could be selected [17, 34], which is a serious limitation given that most studies also find that in most cases there are multiple overlapping motivations (Table 3). Other studies asked an open‐ended question about the (main) motivations for the decision, which were thematically coded into categories by the researchers, in order to use these data in quantitative analyses [16, 31].

Ten selected studies used primarily qualitative methods in the form of in‐depth interviews (Table 2). Some reported having specific topics on their list regarding motivations for the unwanted pregnancy decision [18, 29, 35], while others reported that motivations emerged in topics discussed throughout the entire interview [20, 23, 24, 36].

Seven out of the 19 included studies lacked explicit information on how motivations were measured [20, 21, 22, 23, 24, 26, 27], and in two more studies this information was incomplete [25, 32].

**TABLE 3:** Motivations for abortion or continuation of an unwanted pregnancy in the included studies (*n* = 19).

Reference	Unwanted pregnancy decision	Partner‐related motivations	Material motivations	Motivations related to life circumstances	Motivations related to family planning/child wish	Motivations related to sociocultural pressure, stigma or personal beliefs	Motivations related to the others in the family, friends or social environment	General findings about relations between motivations
Bell et al. [30]	Abortion		Financial resources, financial support, housing	Too young, future plans focused on career or education		Negative experience of pregnancy	Afraid of reaction of others on pregnancy, don't want to have a negative impact on others (family/friends)	Participants gave multiple and complex reasons. Participants who opted for abortion were often able to make this choice quickly, while those who continued the pregnancy had a more difficult and longer decision process
	Continue		Financial resources, financial support, housing	Future plans focused on family, willing to give up other future plans, positive experience with previous pregnancy(s)		Anti‐abortion attitudes; positive experience of pregnancy	Support from others, impact on existing children	
Biggs et al. [35]	Abortion	Unstable romantic relationship	Not financially prepared	Interferes with future opportunities (e.g., study/work), health issues, not prepared, want a better life for the baby	Not the right time for a baby, don't want a baby	Legal issues, fear of giving birth	Influences of family/friends, needs of other children in the family	Authors mention that the reasons womxn seek abortion are complex and interrelated. They also mention that participants generally were not able to narrow their answer to one reason and sometimes even gave additional reasons to this last question, making it difficult to discern a “main” reason
Biney and Atiglo [16]	Abortion	(Too) little support of partner, unstable romantic relationship	Financial constraints	Too old/young, cannot be combined with study/work, health issues	Wanting to space or delay birth		Lack of support (e.g., no partner, to avoid shame, afraid of opinion parents)	Authors mention that womxn usually have more than one reason for an abortion, indicating the multidimensionality of abortion decision‐making
Brauer et al. [28]	Abortion	Unstable romantic relationship	Financial/ material matters (as part of negative life circumstances)	Living conditions not suitable for raising a child		Personal belief: Abortion as a womxn's right	In general, the partner is in favor of having an abortion	Authors mention that participants often cannot mention one decisive factor; decision‐making is a complex process
	Continue	The potential role of the “father” is most important for considering an abortion	Financial/ material matters (as part of negative life circumstances), but those can be overcome	Living conditions not suitable for raising a child, but those can be overcome		Personal belief: Abortion only allowed in extreme situations, afraid of consequences; positive feelings	Opinion of significant others (partner, friends, professionals) is important in decision making	
Chunuan et al. [27]	Abortion	Relationship problems, pregnant while unmarried, boyfriend/ partner does not accept the pregnancy	Financial reasons (insufficient income, debts)	Too old/young, cannot be combined with study/work	Already have enough children, not ready for a baby		Fear of family members' embarrassment about the pregnancy	
Ekstrand et al. [25]	Abortion	Unstable romantic relationship	Not economically realistic to raise a child (unstable income)	Cannot be combined with study/work	Not ready for a baby		Reactions from partners, parents and peers were negative toward a continuation of the pregnancy	Authors mention that an abortion decision (of teenage womxn) often involves conflicting and contradictory emotions
Hosseini‐Chavosh et al. [21]	Abortion	Most abortions took place after the partner agreed to it	Socioeconomic reasons (lack of space in the house)	Health problems		Sociocultural reasons (older womxn are ashamed); religion: abortion as sinful, barrier in decision for abortion	Negative reactions on pregnancy of extended family, needs of other children in the family	
Jones et al. [24]	Abortion		Unable to provide conditions such as stable and loving families, financial security, and a high level of care and attention	Living conditions not suitable for raising a child, emotional difficulties	Timing not right	Adoption not a realistic option	The inability to have another child without compromising the care for existing children	Authors mention that womxn have abortions for manifold and complex reasons
Kirkman et al. [36]	Abortion	Unstable romantic relationship, partner not involved (enough), pregnant after rape, abusive relationship	No economic capacity to provide adequately for the potential child, unstable housing	Life circumstances: Too young, mental problems, wanting to do other things first	Not prepared for motherhood	Not wanting to contribute to the world's overpopulation	Not wanting to put harm on parents or extended family, also considering the needs of existing children	Reasons are categorized as related to the woman herself, the potential child, the sexual partner, existing children, the extended family, financial reasons, and other reasons; which are part of explanatory accounts and are mutually influential. Womxn's accounts reveal the complex personal and social contexts within which they make reproductive decisions and the thoughtfulness with which they make them
Mahanaimy and Moseson [29]	Abortion	Unstable romantic relationship, and/or violent relationship	Unstable housing situation, job and/or finances				Not having enough social support from person involved in the pregnancy, from family members, or from other sources	Authors mention that “a myriad of factors affected the decision”
	Continue	Stable romantic relationshhip		Positive life circumstances (e.g., age, education)		Opposition to abortion from the participant and their friends, and misinformation about abortion	Social support from others	
Makenzius et al. [32]	Abortion	Relationship problems, unstable romantic relationship	Unemployment, poor economy, uncertain housing	Health issues of woman or her partner	Unplanned pregnancy, already had the number of children they wished for	Cultural factors that do not allow children before marriage		Authors mention that some womxn reported multiple reasons. The most mentioned reason was that the pregnancy was unplanned and untimely, followed by partner‐related reasons and socioeconomic factors
Motaghi et al. [22]	Abortion	Pregnancy while unmarried (e.g., during engagement period, or as a widow)	Economic factors (high costs of living)	Too old, cannot be combined with study/work	Having other (young) children (birth spacing), completed their desire to have children, not ready for (another) baby	Afraid of social stigma, and personal beliefs		
Pereira et al. [33]	Abortion		Financial reasons	Cannot be combined with study/work	Wants no (more) children	Womxn who immediately know that they want an abortion often mention that they cannot meet the responsibilities of motherhood		The decision to have an abortion results from a sequence of different decisions and behaviors, which lead to different trajectories, and is motivated by a different combination of reasons
Pestvenidze and Stray‐Pedersen [17]	Abortion		Socioeconomic concerns	Health issues	Wants no (more) children, wants to space childbearing			The most important reason was “did not want any more children” (49%), socioeconomic reasons (22%) and spacing pregnancies (18%). The authors mention that it was a limitation that participants could only choose one reason, which does not fully capture complex motives
Ranji 2012 [31]	Abortion	Husband compelled the abortion, marriage may end soon	Family economic problems	Having a baby would interfere with education, health issues	Wants no (more) children; wants to postpone childbearing			Poverty is not the main reason for abortion in Iran; rather, parents wish to control their family size
Rehnströhm Loi et al. [23]	Abortion	Lack of support from partners, fearing anger, violence, divorce	Financial inability to raise a child	Perceived lack of options: Cannot be combined with study/work, health issues, combined with feelings of shame		Social pressure/stigma associated with mistimed pregnancy (outside of marriage), gender based norms	Disagreement between partners	Silence, secrecy and deep‐rooted stigma around abortion act as driving forces for unsafe abortions
Rowe et al. [34]	Abortion	New or unstable relationship, relationship problems and/or violent relationship	Financial reasons	Too young, medical reasons	Does not want children now, already enough children, not the right time, has a young baby (birth spacing)			Authors mention that the motivations related to family planning/child wish are the most important motivations
Serret and Pairo [20]	Abortion	Relationship status and joint parenthood project: unstable romantic relationship	Financial/ employment situation	Fulfilling aspirations, waiting for more stability, reinforcing personal situations	Lack of desire to be a mother	Social norms: an unwanted pregnancy is irresponsible	Lack of social network to support a new mother	Whether or not the pregnancy continues once an unintended pregnancy has occurred, the dimensions considered by young adult womxn are the same. However, the interplay of factors contributing to a decision is more complex based on their current life stage. Relationship quality is most important, the other reasons less so
	Continue	Stable romantic relationship				Experienced social pressure to become a mother		
Thapa et al. [26]	Abortion	Unmarried, rape (but not mentioned a lot)	Financial reasons; currently unable to afford a child, may be in the future	Not the right time due to other reasons	Desire not to have (more) children, current child too young (birth spacing)	Unmarried (social stigma)		Majority does not want any more children (3/5), or cites finances (2/5). Decisions were often made jointly with partners

### 3.3 People's Motivations to Have an Abortion or to Continue an Unwanted Pregnancy

Of the 19 included articles, 12 demonstrated that multiple factors played a role in the decision to have an abortion or to continue an unwanted pregnancy [16, 20, 24, 25, 28, 29, 30, 33, 35]. The motivations were often interdependent [20, 28, 35, 36] and sometimes even conflicting [25]. In Table 3 we provide an overview of the motivations found in the included studies, giving some insight into the motivations that people share about their unwanted pregnancy decision, and general findings about relations between motivations. Since researchers categorized motivations differently across studies, the overlap and interrelatedness between motivations is reflected in the categorization we used in Table 3. The categorization is not intended to be rigid or mutually exclusive but aims to provide a framework for understanding the diverse motivations discussed in the included studies. Note that some studies also mentioned which motivations were more prevalent than others, and that Table 3 does not fully capture the differences in prevalence for the different motivations. In the following paragraphs, we discuss the most prevalent motivations. Reported motivations are mentioned in studies conducted in countries with different socioeconomic contexts and abortion legality, unless explicitly reported otherwise.

Five studies showed that family planning was the primary motivation for abortion [17, 26, 31, 32, 34]. This was often sub‐categorized into more motivations, such as having a complete family, no desire for children (yet), or not the right time for having a(nother) child. However, not all included studies reported motivations related to family planning (Table 3). For instance, people who continued an unwanted pregnancy did not report family planning motivations, yet they often reported motivations to continue the pregnancy despite negative circumstances related to family expansion [28].

Studies frequently reported various motivations related to the partner involved in the pregnancy (Table 3). For instance, an unstable, unsupportive, absent, or even violent partner relationship was mentioned as a motivation for abortion [16, 20, 23, 25, 28, 29, 32, 34, 35, 36]. At the same time, the partner relationship often contributed to the decision to continue the pregnancy, for example when the partner was supportive, and the relationship was stable [20, 28, 29].

Social support from others in the direct social environment (such as the family) also contributed to the decision to have an abortion or to continue an unwanted pregnancy (Table 3). Studies mentioned that strong social support was a motivation to continue an unwanted pregnancy [29, 30], whereas a lack of social support came up as a motive for abortion [17, 20, 29].

Further, research showed that some people strongly valued the opinions of others. Some seemed afraid that the pregnancy would negatively impact others [30, 36], and therefore chose to have an abortion. The (expected) opinion of others (such as the partner involved in the pregnancy, family or friends) was an important motive for both the decision to have an abortion or to continue an unwanted pregnancy [16, 21, 25, 27, 28, 35].

All 19 studies mentioned material motivations. These were primarily associated with inadequate financial resources, such as an unstable employment situation or lack of space in the home (Table 3). In contrast, only two studies in HICs (out of four studies investigating continued unwanted pregnancies) reported material motivations for continuing an unwanted pregnancy, such as financial support [20], or practical concerns that could be overcome [28]. Notably, no studies mentioned material circumstances as the only motivation for abortion or for continuing an unwanted pregnancy.

Almost all studies on motivations for both abortion and continuing an unwanted pregnancy mentioned life circumstances (Table 3). For instance, people mentioned being occupied with work or study, being (too) old or young, or having health concerns as motivations that influence the decision for abortion [16, 17, 20, 21, 22, 23, 24, 25, 27, 30, 31, 32, 33, 34, 35, 36]. People also considered the impact on existing children [21, 24, 26, 30, 36], and some studies explicitly noted that the needs of existing children motivated the decision to have an abortion (such as needing more personal attention) [24, 36]. On the other hand, people mentioned positive life circumstances, such as having the right age or having finished one’s education, as motives to continue an unwanted pregnancy [29]. People who continued an unwanted pregnancy also often stated that they could overcome their negative life circumstances [28], or that they were willing to give up other future plans [30].

Furthermore, studies identified social stigma as a motivation for abortion (Table 3). Some of this stigma related to unwanted pregnancy, such as the stigma associated with being pregnant outside of marriage [23, 26, 32]. Other people experienced the stigma that an unwanted pregnancy is the consequence of irresponsible behavior [20]. People identified the stigma of abortion as a barrier in the decision to have an abortion. For instance, people stated that they felt abortion was sinful [21]. A Nepalese study [23] found that silence, secrecy and stigma were the driving forces for people to have unsafe abortions, despite abortion being legal. On the other hand, people continuing an unwanted pregnancy mentioned that they experienced social stigma and pressure to become a mother, which influenced their decision to continue the unwanted pregnancy to term [20].

Personal experiences and beliefs about pregnancy and abortion influence both the decision to have an abortion or to continue an unwanted pregnancy. Having positive feelings toward the pregnancy or previous pregnancies [28, 30], as well as the fear of negative consequences of abortion sometimes influenced the decision to continue an unwanted pregnancy [28, 29, 30]. These four studies on continued unwanted pregnancies [20, 28, 29, 30] were all conducted in countries where abortion is allowed on request with varying gestational limits. Conversely, these personal experiences and beliefs may have also influenced the decision for abortion: people stated that they feared giving birth or had other negative experiences with previous pregnancies [30, 35].

## 4 Discussion and Conclusions

In public and political debates, people often raise the questions: Why do people have abortions? And why do others choose to carry an unwanted pregnancy to term? The research we reviewed showed that in different high, middle and low income countries with varying levels of access to abortion care and even in situations where abortion is prohibited, explanatory accounts of abortion decisions are mostly multifactorial and interrelated. Despite variations in methods and their limitations, studies onabortion or continuation of unwanted pregnancies revealed this consistent picture of decisions that are influenced by a range of interrelated motives. This aligns with previous reviews of the literature [6, 7], but in the current study, we extended these findings to even more diverse contexts, such as countries with restrictive abortion policies, and to unwanted pregnancies that people decided to continue.

Womxn's accounts of their motivations for abortion or continuation of an unwanted pregnancy seem relatively universal to some extent, but they also differ between countries. Almost all studies mentioned motivations such as family planning/desire to have a child, life circumstances, the partner relationship, and material circumstances. Motivations related to the sociocultural environment, such as experienced stigma or shame of getting pregnant unintentional (e.g., getting pregnant out of wedlock), or anti‐abortion narratives (e.g., abortion is sinful or only allowed in very extreme situations), were not always mentioned, but appeared more often in countries with restrictive abortion policies [16, 21, 22, 23, 27]. Some studies in countries with more liberal abortion policies also mentioned these sociocultural motivations, but to a lesser extent [28, 30].

We found both commonalities and differences in motivations for abortion or continuation of an unwanted pregnancy. In both situations, motivations often related to the partner involved in the pregnancy, social support, and to personal beliefs around abortion or having children in the current situation (e.g., out of wedlock). The dimensions considered seemed to be the same [18, 21], yet people interpreted them in a different way. People often described motivations to continue a pregnancy as counter motivations to why the pregnancy was unwanted. On the other hand, people continuing an unwanted pregnancy hardly ever mentioned family planning motivations, whereas people choosing abortion often mentioned them. We cautiously interpreted the results for pregnancies carried to term, since only four out of the 19 studies included them, and these studies were all conducted in countries with relative liberal abortion laws [20, 28, 29, 30].

When interpreting our findings, it is important to note that the results mostly show explanatory accounts of decision making, and may not reflect the most important aspects that drove the decision making—even though they are more informative then social determinants or correlates of unwanted pregnancy outcomes. People may perceive asking about motivations as asking for a “justification” of a decision [36]. Since abortion is stigmatized, restricted or even banned in many countries [3], people may mention more common motivations because they perceive them as more legitimate or acceptable. It may be impossible to separate people's motivations for unwanted pregnancy decisions from their desire to justify those, since justification may also be directed toward oneself [6]. Moreover, rationalizing how a person arrives at a decision might not align with the actual internal decision‐making process regarding an unwanted pregnancy, which is also driven by emotions and intuitions [2]. We therefore seriously question the frequency and order in which studies mention motivations. Lastly, it is important to stress that although findings show that motivations are multidimensional and interrelated, this does not mean that unwanted pregnancy decision‐making is always inherently “difficult.” Womxn often know immediately what to do after finding out they are pregnant unintendedly [28]. Describing the decision process as “complex” aligns with how researchers experience it, but does not reflect the lived experience of all people: some find the decision quite uncomplicated [37, 38]. Although experiences of negative coping after abortion exist [39], research shows that at least within HICs, most people are satisfied with their decision, irrespective of the motivation, and remain so over time [37, 40, 41].

We should note that research on this topic has limitations, some of which are inherent to this field. Unfortunately, half of the selected studies did not explain how they measured motivations. Another limitation related to the retrospective nature of asking about motivations for decisions already made, which are not necessarily rational choices. For many people, the decision to have an abortion or continue an unwanted pregnancy is not a matter of choice, but an inevitable outcome shaped by personal beliefs, sociocultural, or structural factors. Characterizing this as a rational “weighing process” misrepresents the lived experiences of many people and ignores structural inequalities. An additional limitation of retrospective reporting is that respondents may (intentionally) try to justify their decision, or unintentionally report motivations that did not exist before they made their decision. Future studies should accurately report their measures and/or topic lists. We also recommend that future studies focus more on the process of decision‐making rather than on the outcome of pregnancy decisions, as seen in other research exploring people's narratives behind their abortion decisions [2]. This approach would allow for a deeper understanding of the decision‐making process, rather than asking individuals to rationalize their choices. Lastly, the search parameters for this review did not find any studies investigating the perspective of the partner involved in the pregnancy. More research on the partner's perspective is needed, especially given the importance of the partner relationship as a motivation for abortion or to continue an unwanted pregnancy [42].

This scoping review had some limitations. Scoping reviews are typically broad at the expense of depth, and our study is no exception. We could only investigate the value of included research to a limited extent because half of included studies did not provide details on how they measured motivations. Second, the results for motives for continued unwanted pregnancies may not be generalizable, since we included four studies about these only, all conducted in high income countries. Third, our narrow search strategy—focusing specifically on terms like “motivations”, “reasons”, and “decisions”—probably excluded other valuable literature on the decision‐making narratives of individuals with unwanted pregnancies, such as those included in the recent review of Dalmijn et al. [2]. Lastly, despite frequently reflecting on our positionality and discussions with the advisory committee, we cannot completely exclude the influence of the homogenous composition of our research team. Furthermore, we deliberatly aimed to respect the diverse lived experiences of individuals choosing either an abortion or continuation of an unwanted pregnancy, but the reviewed studies may have reflected legal or societal norms that prioritized certain motivations over others.

In conclusion, this scoping review shows that across various global settings – from high to low income countries, with free access to abortion to highly restricted abortion care ‐ multifactorial and interrelated motivations play a role in a decision to either have an abortion or continue an unwanted pregnancy. Furthermore, it also highlights the difficulties researchers face in gaining a good insight into motivations. In line with Kirkman et al. [6], the current study emphasizes that qualitative research, where respondents are encouraged to describe their actual experiences as well as feelings, instead of being explicitly asked “why”, provides more insight into the actual decision making process. By connecting more closely with personal experience, researchers and professionals can gain more insight into the elements involved in decision‐making that are missed when asking about “reasons.”

## Supporting information


**Table S1a.**Search strategy in PubMed.
**Table S1b.**Search strategy in Web of Science.
**Table S1c.**Search strategy in Scopus.
**Table S1d.**Search strategy in PsychInfo.
**Table S2a.**Study quality of included interview studies (*n* = 10), as assessed by the Critical Appraisal Skills Programme (CASP) for qualitative studies.
**Table S2b.**Study quality of included survey studies (*n* = 9), as assessed by the Critical Appraisal Skills Programme (CASP) for cohort studies.

## Endnotes

^1^
This also includes transgender men and nonbinary individuals with the capacity for pregnancy. Since we are mostly referring to previous research in which no other gender categories are mentioned but women, we will use the term womxn throughout this article.
