# Supplementary material for: Motivations for Abortion or Continuation of an Unwanted Pregnancy: A Scoping Review of the Global Literature
Source: Perspect Sex Reprod Health. 2025 Jan 22;57(1):45–62. doi: 10.1111/psrh.12293 (PMC11936852; doi:10.1111/psrh.12293)
Supplement: Supplementary file 1 — Table S1a.Search strategy in PubMed. Table S1b.Search strategy in Web of Science. Table S1c.Search strategy in Scopus. Table S1d.Search strategy in PsychInfo. Table S2a.Study quality of included interview studies (n = 10), as assessed by the Critical Appraisal Skills Programme (CASP) for qualitative studies. Table S2b.Study quality of included survey studies (n = 9), as assessed by the Critical Appraisal Skills Programme (CASP) for cohort studies. [file PSRH-57-45-s001.docx]

Supplementary files

# Supplementary table 1a. Search strategy in PubMed.

| **Search** | **Query** | **Items found** |
| --- | --- | --- |
| #1 | Pregnancy, unplanned [MeSH terms] OR pregnancy, unwanted [MeSH terms] OR Unintended pregnancy [tiab] | 6,948 |
| #2 | Motiv [tiab] OR Reasons [tiab] OR Explanation [tiab] OR Motivation [MeSH terms] | 513,917 |
| #3 | Decision making [MeSH terms] | 231,396 |
| #4 | Abortion, Induced / psychology [MeSH terms] OR termination of pregnancy [tiab] OR Parenting [tiab] OR Pregnancy outcomes [tiab] | 56,167 |
| #5 | #1 AND #2 | 785 |
| #6 | #1 AND #2 AND #3 | 67 |
| #7 | #1 AND #2 AND #3 AND #4 | 27 |
|  | Articles since 2008 | 19 |

# Supplementary table 1b. Search strategy in Web of Science.

| **Search** | **Query** | **Items found** |
| --- | --- | --- |
| #1 | TS=(unplanned pregnancy OR unwanted pregnancy OR Unintended pregnancy ) | 11,583 |
| #2 | TS=(Motiv OR Reasons OR Explanation) | 1,064,122 |
| #3 | TS=(Decision making) | 629,109 |
| #4 | TS=(Abortion OR termination of pregnancy OR Parenting OR Pregnancy outcomes) | 245,118 |
| #5 | #1 AND #2 | 1,028 |
| #6 | #1 AND #2 AND #3 | 98 |
| #7 | #1 AND #2 AND #3 AND #4 | 62 |
|  | Articles since 2008 | 54 |

# Supplementary table 1c. Search strategy in Scopus.

| **Search** | **Query** | **Items found** |
| --- | --- | --- |
| #1 | TITLE-ABS-KEY ( unintended AND pregnancy OR unplanned AND pregnancy OR unwanted AND pregnancy ) | 6,252 |
| #2 | TITLE-ABS-KEY(Reasons OR Motivation OR Explanation) | 1,989,981 |
| #3 | TITLE-ABS-KEY (Decision AND Making) | 1,093,333 |
| #4 | TITLE-ABS-KEY (termination AND of AND pregnancy OR Pregnancy AND outcomes OR Abortion OR Parenting) | 14,762 |
| #5 | #1 AND #2 | 638 |
| #6 | #1 AND #2 AND #3 | 70 |
| #7 | #1 AND #2 AND #3 AND #4 | 26 |
|  | Articles since 2008 | 20 |

# Supplementary table 1d. Search strategy in PsychInfo.

| **Search** | **Query** | **Items found** |
| --- | --- | --- |
| #1 | (Unintended pregnancy or Unplanned pregnancy or Unwanted pregnancy).mp | 2,372 |
| #2 | (Reasons or Motivation or Explanation).mp | 291,719 |
| #3 | Decision Making.mp. or Choice Behavior/ | 165,571 |
| #4 | (termination of pregnancy or Pregnancy outcomes or ("Abortion (Induced)" or Induced Abortion) or Parenting).mp | 64,832 |
| #5 | #1 AND #2 | 183 |
| #6 | #1 AND #2 AND #3 | 30 |
| #7 | #1 AND #2 AND #3 AND #4 | 12 |
|  | Articles since 2008 | 9 |

**Supplementary table 2a.** Study quality of included interview studies (*n*  = 10), as assessed by the Critical Appraisal Skills Programme (CASP) for qualitative studies.

|  | **Rehnströhm et al. (2019)** | **Serret & Pairo (2018)** | **Motaghi et al. (2013)** | **Kirkman et al. (2010)** | **Hosseini-Chavosh, et al. (2012)** | **Brauer et al. (2019)** | **Jones et al. (2008)** | **Ekstrand et al. (2009)** | **Mahanaimy & Moseson (2022)** | **Biggs, Gould & Foster (2013)** |
| --- | --- | --- | --- | --- | --- | --- | --- | --- | --- | --- |
| **Was there a clear statement of the aims of the research?** | Yes | Yes | Yes | Yes | Yes | Yes | Yes | Yes | Yes | Yes |
| **Is a qualitative methodology appropriate?** | Yes | Yes | Yes | Yes | Yes | Yes | Yes | Yes | Yes | Yes |
| **Was the research design appropriate to the aims of the research?** | Yes | Yes | Yes | Yes | Yes | Yes | Yes | Yes | Yes | Yes |
| **Was the recruitment strategy appropriate to the aims of the research?** | Yes, but  not reported how many participants did not want to participate (and why). | Yes | Yes, but not reported how many participants did not want to participate (and why). | Yes, but not reported how many participants did not want to participate (and why). | It is not clear how the 40 women were selected for the in-depth interviews. | Yes | Yes | Yes | Yes | Yes |
|  | **Rehnströhm et al. (2019)** | **Serret & Pairo (2018)** | **Motaghi et al. (2013)** | **Kirkman et al. (2010)** | **Hosseini-Chavosh, et al. (2012)** | **Brauer et al. (2019)** | **Jones et al. (2008)** | **Ekstrand et al. (2009)** | **Mahanaimy & Moseson (2022)** | **Biggs, Gould & Foster (2013)** |
| **Were the data collected in a way that  addressed the research issue?** | Not much info about ways 'reasons' were discussed in the interview. Topic list etc. is missing. | Not much info about ways 'reasons' were discussed in the interview. Topic list etc. is missing. | Not much info about ways 'reasons' were discussed in the interview. Topic list etc. is missing. | Yes | No report about what kind of topics were discussed during the interview. | Yes | Not much info about ways 'reasons' were discussed in the interview. Topic list etc. is missing. | Yes | Yes | Yes |
| **Has the relationship between researcher and participants been adequately considered?** | Yes | No info given on back-ground of researchers and no consider-  ation of possibility of researcher bias. | No info given on back-ground of researchers and no consider-  ation of possibility of researcher bias. | Yes | No info given on back-ground of researchers and no consider-  ation of possibility of researcher bias. | Yes | No info given on back-ground of researchers and no consider-  ation of possibility of researcher bias. | No info given on back-ground of researchers and no consider-  ation of possibility of researcher bias. | Yes | Yes |
|  | **Rehnströhm et al. (2019)** | **Serret & Pairo (2018)** | **Motaghi et al. (2013)** | **Kirkman et al. (2010)** | **Hosseini-Chavosh, et al. (2012)** | **Brauer et al. (2019)** | **Jones et al. (2008)** | **Ekstrand et al. (2009)** | **Mahanaimy & Moseson (2022)** | **Biggs, Gould & Foster (2013)** |
| **Have ethical issues been taken into consideration ?** | Yes | Nothing reported about ethical approval. | Yes | Yes | Nothing reported about ethical approval. | Yes | Yes, however not much info about confi-dentiality of in the interviewprotocol. | Yes | Yes | Yes |
| **Was the data analysis sufficiently rigorous?** | Yes | Yes | Yes | Yes | Yes | Yes | Yes | Yes | Yes | Yes |
| **Is there a clear statement of  findings?** | Yes | Yes | Yes | Yes | Yes | Yes | Yes | Yes | Yes | Yes |
| **How valuable is the research?** | Valuable. | Valuable. | Valuable,  but not discussed what the study adds to literature, and no insights into generali-zability of results. | Valuable. | Valuable, but not discussed what the study adds to literature, and no insights into generali-zability of results. | Valuable. | Valuable. | Valuable. | Valuable. | Valuable. |

**Supplementary table 2b.** Study quality of included survey studies (*n*  = 9), as assessed by the Critical Appraisal Skills Programme (CASP) for cohort studies.

|  | **Thapa et al. (2018)** | **Rowe et al. (2009)** | **Ranji (2012)** | **Pestvenidze & Stray-Pedersen (2018)** | **Pereira, Pires & Canavaro (2019)** | **Chunuan et al. (2012)** | **Bell et al. (2013)** | **Biney et al. (2017)** | **Makenzius et al.**  **(2011)** |
| --- | --- | --- | --- | --- | --- | --- | --- | --- | --- |
| **Did the study address a clearly focused issue?** | Yes | Yes | Yes | Yes | Yes | Yes | Yes | Yes | Yes |
| **Was the cohort recruited in an acceptable way?** | Yes | Yes | Yes | Yes | Yes | Yes | Yes | Yes | Yes |
| **Were 'reasons' for pregnancy decision outcome accurately measured to minimise bias?** | Not sure; No info about what questions were used. | Yes; Drop-down menu with most important reasons (categories). | Yes; Open-ended question. Answers were categorized. | Yes; Categorical question, but it is not clear how question was phrased. | Yes; The Reasons for Abortion List (RAL) was used. | Not sure; Not clear what was asked about reasons for abortion. | Yes; Open-ended question. Answers were categorized. | Yes; Open-ended question. Answers were categorized | Yes; Open-ended question. Answers were categorized. |
| **Have ethical issues been taken into consideration ?** | Yes | Yes | Yes | Yes | Yes | Yes | Yes | Nothing reported about ethical considerations. | Yes |
|  | **Thapa et al. (2018)** | **Rowe et al. (2009)** | **Ranji (2012)** | **Pestvenidze & Stray-Pedersen (2018)** | **Pereira, Pires & Canavaro (2019)** | **Chunuan et al. (2012)** | **Bell et al. (2013)** | **Biney et al. (2017)** | **Makenzius et al.**  **(2011)** |
| **Are the results precise?** | Yes | Yes | Yes | Yes | Yes | Yes | Yes | Yes | Yes |
| **Can the results be applied to the local population?** | Yes | Yes | Yes | Yes | Yes | Yes | Yes | Yes | Yes |
